# Insight into the antibacterial resistance of graphdiyne functionalized by silver nanoparticles

**DOI:** 10.1111/cpr.13236

**Published:** 2022-05-03

**Authors:** Simin Qin, Mo Xie, Shuting Cao, Jiang Li, Lihua Wang, Shi‐Hua Luo, Min Lv

**Affiliations:** ^1^ Division of Physical Biology, CAS Key Laboratory of Interfacial Physics and Technology Shanghai Institute of Applied Physics, Chinese Academy of Sciences Shanghai China; ^2^ University of Chinese Academy of Sciences Beijing China; ^3^ State Key Laboratory of Organic Electronics and Information Displays & Jiangsu Key Laboratory for Biosensors, Institute of Advanced Materials (IAM), Jiangsu National Synergetic Innovation Center for Advanced Materials (SICAM) Nanjing University of Posts and Telecommunications Nanjing China; ^4^ The Interdisciplinary Research Center, Shanghai Synchrotron Radiation Facility, Zhangjiang Laboratory Shanghai Advanced Research Institute, Chinese Academy of Sciences Shanghai China; ^5^ Department of Traumatology, Rui Jin Hospital, School of Medicine Shanghai Jiao Tong University Shanghai China; ^6^ College of Chemistry and Materials Science Shanghai Normal University Shanghai China

## Abstract

**Objectives:**

Silver nanoparticles (AgNPs) tend to aggregate spontaneously due to larger surface‐to‐volume ratio, which causes decreased antibacterial activity and even enhanced antimicrobial resistance (AMR). Here, we aim to improve the stability of AgNPs by employing a growth anchor graphdiyne (GDY) to overcome these shortcomings.

**Materials and Methods:**

*Bacillus subtilis* and *Escherichia coli* were selected to represent gram‐positive and gram‐negative bacteria, respectively. Transmission electron microscopy (TEM), energy dispersive spectroscopy (EDS), scanning electron microscopy (SEM)‐EDS mapping and inductively coupled plasma mass spectrometry (ICP‐MS) were carried out to characterize the physiochemical properties of materials. The antimicrobial property was determined by turbidimetry and plate colony‐counting methods. The physiology of bacteria was detected by SEM and confocal imaging, such as morphology, reactive oxygen species (ROS) and cell membrane.

**Results:**

We successfully synthesized a hybrid graphdiyne @ silver nanoparticles (GDY@Ag) by an environment‐friendly approach without any reductants. The hybrid showed high stability and excellent broad‐spectrum antibacterial activity towards both gram‐positive and gram‐negative bacteria. It killed bacteria through membrane destruction and ROS production. Additionally, GDY@Ag did not induce the development of the bacterial resistance after repeated exposure.

**Conclusions:**

GDY@Ag composite combats bacteria by synergetic action of GDY and AgNPs. Especially, GDY@Ag can preserve its bacterial susceptibility after repeated exposure compared to antibiotics. Our findings provide an avenue to design innovative antibacterial agents for effective sterilization.

## Graphical abstract

Graphdiyne@silver nanoparticles (GDY@Ag) composite preserves its bacterial susceptibilities after repeated exposure compared to antibiotics.

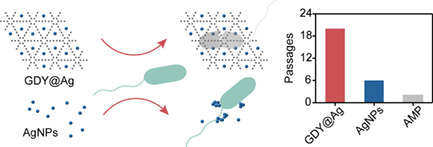

## 1 INTRODUCTION

Antimicrobial resistance (AMR) is an imminent threat to global public health and national wealth.^1^ It is estimated that AMR will cause 10 million deaths a year by 2050 without intervention.^2^ These resistant bacterial infections would exact a US$3.4 trillion health‐care cost by 2030.^3^ Antibiotics are routinely used as therapeutics for clinical infections since the discovery of penicillin. These antimicrobials combat bacteria by interacting with special intracellular targets via chemical ways, which easily induce the resistance in bacteria.^4^ What is worse, the horizontal gene transfer between bacteria can further accelerate the propagation of resistance, causing the novel antibiotics quickly to fail.^5^, ^6^ Therefore, there is an urgent need to develop novel alternatives to combat the growing menace of pathogenic bacteria.

Silver‐based materials have been used as disinfectants empirically for a long history. In the last few decades, various forms of nano‐silver species were widely used as antibacterial components in coatings,^7^ wound dressings^8^, ^9^ and other products,^10^, ^11^, ^12^ due to their high bactericidal efficiency. Nevertheless, silver is not always effective, for resistance to ionic silver has been acknowledged for years.^13^, ^14^, ^15^ A recent study even has reported that three gram‐negative bacteria can adapt to silver nanoparticles (AgNPs) exposure by producing flagellum protein to induce aggregation of AgNPs.^16^ In addition to the passive aggregation of AgNPs causing bacterial resistance in this study, the spontaneous agglomerated AgNPs exhibit weakened antibacterial activity, which has been well‐established in a lot of literature.^17^, ^18^ To our knowledge, silver nanoparticles tend to agglomerate in media due to their larger surface‐to‐volume ratio and higher surface energy.^19^ Hence, it is critical to control the stability of AgNPs to maintain their excellent antibacterial activity in practical application. Significant efforts have been made to stabilize Ag nanoparticles by employing an anchor for its growth, such as polymers,^20^ silicon‐based materials^9^ and carbon‐based materials.^21^ Amongst them, carbon nanomaterials with large conjugated π‐systems and abundant anionic functional groups (e.g., carboxyl, hydroxy and epoxy groups) have been considered as an ideal surface for the in situ growth of Ag nanoparticles by reduction reaction.^17^, ^18^, ^21^

Graphdiyne (GDY) is a novel two‐dimensional (2D) carbon allotrope,^22^ consisting of 18‐C hexagon precursors with sp^2^‐hybridized benzene rings and sp‐hybridized alkyne groups connected by butadiyne linkage (–C ≡ C–C ≡ C–). The well‐distributed triangular pores exhibit higher affinity with molecules, ions and compounds. Especially, the plane π/π* states strengthen the combination between metal atoms and alkynyl,^23^, ^24^ endowing GDY an ideal substrate for metal nanoparticles. Various GDY metal or metal oxide nanocomposites have been reported,^24^ for example, Ni/graphdiyne and Fe/graphdiyne,^23^ palladium‐iron nanostructure‐coated graphdiyne nanosheet (PdFe/GDY),^25^ TiO_2_/graphdiyne composites (TiO_2_/GDY)^26^ and so on. GDY‐based nanomaterials show specific electric property, high catalytic activity, increased stability and biocompatibility, which have attracted considerable attention in biomedical applications including biosensor, cancer treatment and clinical infection therapeutics. For example, they have emerged as a new nanozyme with increased intrinsic catalytic capacities to kill bacteria by producing highly toxic radicals.^25^, ^27^, ^28^ The disadvantage of nanozyme‐related antibacterial strategy is that the reaction mostly requires additional hydrogen peroxide to trigger. In fact, Li's group have demonstrated that GDY and graphdiyne oxide (GDYO) can be used as photocatalytic antibacterial agents against bacteria. GDYO with good dispersion in the bacterial system showed stronger antibacterial activity than GDY both in dark and visible light irradiation.^29^ The excellent inhibiting capacity of GDYO against bacteria is attributed to oxidative stress. However, in another study, GDY exhibits higher antimicrobial property than that of GDYO, and “physical” effects play a major role in the antibacterial process.^30^ Although these conclusions are contradictory, it is sure that both GDY and GDYO are capable of hindering broad‐spectrum bacterial growth. Therefore, we postulate that the hybrid of GDY and AgNPs may not only solve the stability of Ag nanoparticles, but also provide a new bactericide with a synergistic antimicrobial effect.

**FIGURE 1:**
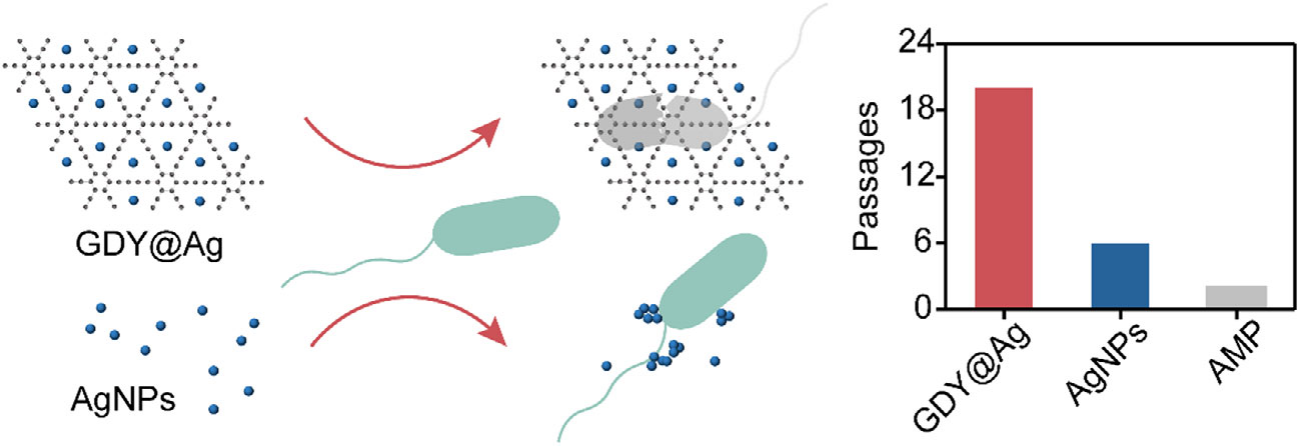
Schematic of stable GDY@Ag with increased antimicrobial property and bacterial susceptibility compared to unstable AgNPs^16^

In this work, we described a hybrid graphdiyne @ silver nanoparticles (GDY@Ag) as a high‐performance bactericide. GDY@Ag was synthesized by simply mixing silver nitrate with GDY; surfactant was added to assist the growth of nanoparticles. The acetylenic groups in GDY acted not only as a reductant, but also as an anchor for AgNPs growth. The hybrid GDY@Ag showed exceptional broad‐spectrum antibacterial activity towards both gram‐positive and gram‐negative bacteria. Moreover, two bacterial strains did not develop the resistance to GDY@Ag after repeated exposure (Figure 1). Our findings present an avenue to fabricate new antibacterial agents for effective bactericidal activity.

## 2 MATERIALS AND METHODS

### 2.1 Materials

AgNO_3_, agar and chloramphenicol were purchased from China National Medicines Corporation Ltd. Luria‐Bertani (LB) broth and ampicillin were purchased from Sangon Biotech (Shanghai) Co., Ltd. Hexadecyltrimethylammonium bromide (CTAB) was purchased from Sigma‐Aldrich. *Bacillus subtilis* and *Escherichia coli* were purchased from the China General Microbiological Culture Collection Center (CGMCC).

### 2.2 Synthesis and characterization of GDY@Ag

The preparation of GDY powder was carried out according to previous work.^22^ Then, GDY powder was dispersed into ultrapure water and ultrasonically agitated for 12 h to form a homogeneous solution with an ultrasonic cleaner (Kunshan ultrasonic instrument Co., Ltd., KQ‐200KDE, frequency = 40KHz, input power = 200 W). GDY solution (1 mg/ml) was stored at room temperature.

GDY@Ag was prepared by a modified protocol published by Yuliang Li.^31^ In brief, 500 μl of the above GDY solution was mixed with 4 ml of 5‐mM AgNO_3_ aqueous solution under vigorous magnetic stirring (700 rpm/min) at room temperature. After stirring for 10 min, 500 μl of 100‐mM CTAB aqueous solution was added dropwise to the reaction mixture and continuously stirred for 20 min. The products were collected by centrifugation at 13,000 rpm for 15 min, followed by washing with ultrapure water four times.

The sample was dropped onto carbon film‐coated copper grids and absorbed for 5 min and then washed with about 50‐μl ultrapure water dropwise. The morphological structures of GDY and GDY@Ag were observed by transmission electron microscope (TEM) (FEI Tecnai G2 F20 S‐TWIN, USA) and scanning electron microscope (SEM) (LEO 1530vp, Germany).^32^ The energy dispersive spectroscopy (EDS) mapping (Oxford) was performed to analyse the element. The amount of silver element loaded on the GDY@Ag was quantified using an inductively coupled plasma mass spectrometer (ICP‐MS) (NexION 300D, PerkinElmer, USA).

### 2.3 Bacteria culture

*Escherichia coli* (G−) and *Bacillus subtilis* (G+) were employed to evaluate the antibacterial activity of GDY@Ag. The strains were grown overnight in fresh LB medium at 37°C in an incubator whilst shaking (220 rpm/min) and then harvested at the exponential growth phase. The bacterial cells were washed twice and resuspended in sterile saline solution (0.9% NaCl). The bacterial concentration was quantified by measuring the optical density at 600 nm (OD_600_).^33^ The cultures can be stored at 4°C for a short time to be used in further experiments.

### 2.4 Antibacterial activity test

#### 2.4.1 Minimum inhibitory concentration test

The bacteria at the concentration of 1 × 10^7^ CFU/ml were cultured with different concentrations of GDY@Ag in LB medium. The MIC value was defined as the lowest concentration that the suspension was transparent when viewed with naked eyes after 18 h at 37°C in a shaking incubator with a rotation motion at 220 rpm/min. The OD_600_ was measured by a synergy MX H1 microplate reader (Gene Company Limited, China).

#### 2.4.2 Instant and long‐term antibacterial Activity of GDY@Ag

Bacterial cultures were prepared using the same method as the aforementioned MIC test. Samples were collected in designed time points including 0.25, 0.5, 1, 1.5 and 2 h. The mixture was diluted with a gradient method and spread uniformly on three LB agar plates and then incubated at 37°C for 22 h. The colony‐forming units (CFU) were counted. For the long‐term test, bacterial cells were cultivated with different concentrations of GDY@Ag at 37°C for 72 h in a shaking incubator at 220 rpm/min. The samples were collected every 2 h for the first 20 h and every 12 h from the 24th h. Bacterial concentrations of all samples were finally determined by measuring OD_600_.

### 2.5 Bacterial physiology analysis

The bacterial cells were treated with GDY@Ag for 2 h and washed with sterile saline solution by centrifugation at 13,000 rpm. On the one hand, the samples were stained according to the LIVE/DEAD™ BacLight™ Bacterial Viability Kit (Thermo Fisher), and then, 5‐μl stained bacterial suspension was trapped between a slide and a square coverslip. TCS SP8 laser confocal microscope (Leica, Germany) was used to analyse the bacterial survival.^34^ On the other hand, the samples were fixed with the mixture of paraformaldehyde and glutaraldehyde for 3 h and then were dehydrated by gradient ethanol (35%, 50%, 75%, 85%, 95% and 100%) for 10 min each. Subsequently, 10‐μl dehydrated suspension was dropped onto silicon wafers and dried naturally, then sputter coated with gold for SEM imaging. Besides, the samples were stained with fluorescent 2′,7′‐dichlorodihydrofluorescein diacetate (DCFH‐DA) probe to detect the ROS generation.^35^

### 2.6 Bacterial resistance development test

The wild‐type strains were incubated with sub‐MIC of GDY@Ag at 37°C and 220 rpm/min for 24 h; ampicillin and chloramphenicol were used as negative control towards *E. coli* and *B. subtilis*, respectively. The obtained culture was determined in the first passage. The MIC was tested as described above, and the passage was treated with the corresponding sub‐MIC to acquire the second passage; 20 passages were finally obtained in this way; MICs of every passage were recorded.

## 3 RESULTS

### 3.1 Preparation and characterization of GDY@Ag

**FIGURE 2:**
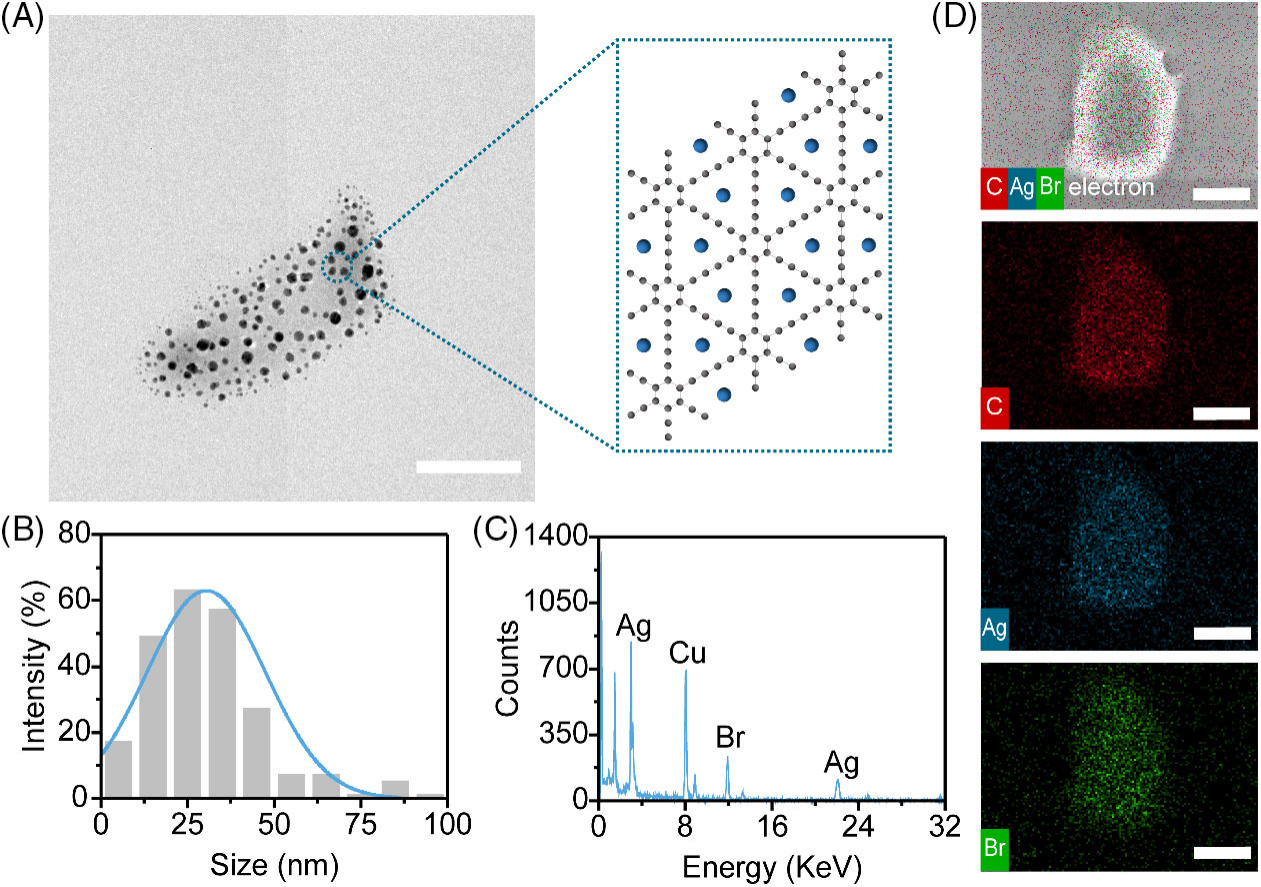
Characterization of GDY@Ag. (a) TEM image of GDY@Ag (Scale bar, 100 nm). (b) Size distribution of AgNPs measured from raw TEM images. Histograms of 232 AgNPs' sizes were normalized and fitted to Gaussian distribution curves (light blue). (c) EDS elemental analysis of GDY@Ag. (d) EDS mapping of GDY@Ag (C is coloured in red, Ag in blue and Br in green, scale bar, 500 nm)

We prepared a hybrid graphdiyne @ Ag nanoparticles (GDY@Ag) according to the previously reported method.^31^ Briefly, silver nitrate as the resource of Ag was mixed with the aqueous GDY solution sufficiently with vigorous stirring. Subsequently, aqueous surfactant CTAB was added to assist the seeding growth of the Ag species.^31^, ^36^ To obtain pure GDY@Ag, the mixture was centrifuged and washed with deionized water for several times to remove residual Ag^+^ and CTAB. Then, we performed transmission electron microscopy (TEM) to show the morphology of GDY (Figure S1) and GDY@Ag (Figure 2a). The images revealed that Ag nanoparticles were randomly anchored on the surface of graphdiyne sheets and the average size was 30.5 ± 17.2 nm (Figure 2b). The EDS confirmed the presence of elemental Ag and Br in the GDY@Ag composite (Figure 2c). Scanning electron microscopy (SEM)‐EDS mapping showed that the Ag element was well overlapped with Br and C elements (Figure 2d). These results demonstrated the uniform adsorption of the Ag nanoparticles on graphdiyne sheets. Br element dissociated from CTAB does exist, indicating that there are also AgBr nanoparticles (AgBrNPs) in GDY sheets.^37^ No signal of N element derived from the quaternary ammonium group of CTAB was observed, suggesting that the local concentration of CTAB was far below the detection limit of 0.1% (w/w%) of EDS mapping. Due to benzene rings and alkyne units arranged in sp‐hybridized atoms and a large conjugated surface, GDY has great advantages in the adsorption and immobilization of metal atoms.^23^, ^38^, ^39^ Our results demonstrated that AgNPs as well as AgBrNPs were immobilized uniformly on the GDY sheets that we successfully prepared GDY@Ag composite.

### 3.2 Antimicrobial property of GDY@Ag

**FIGURE 3:**
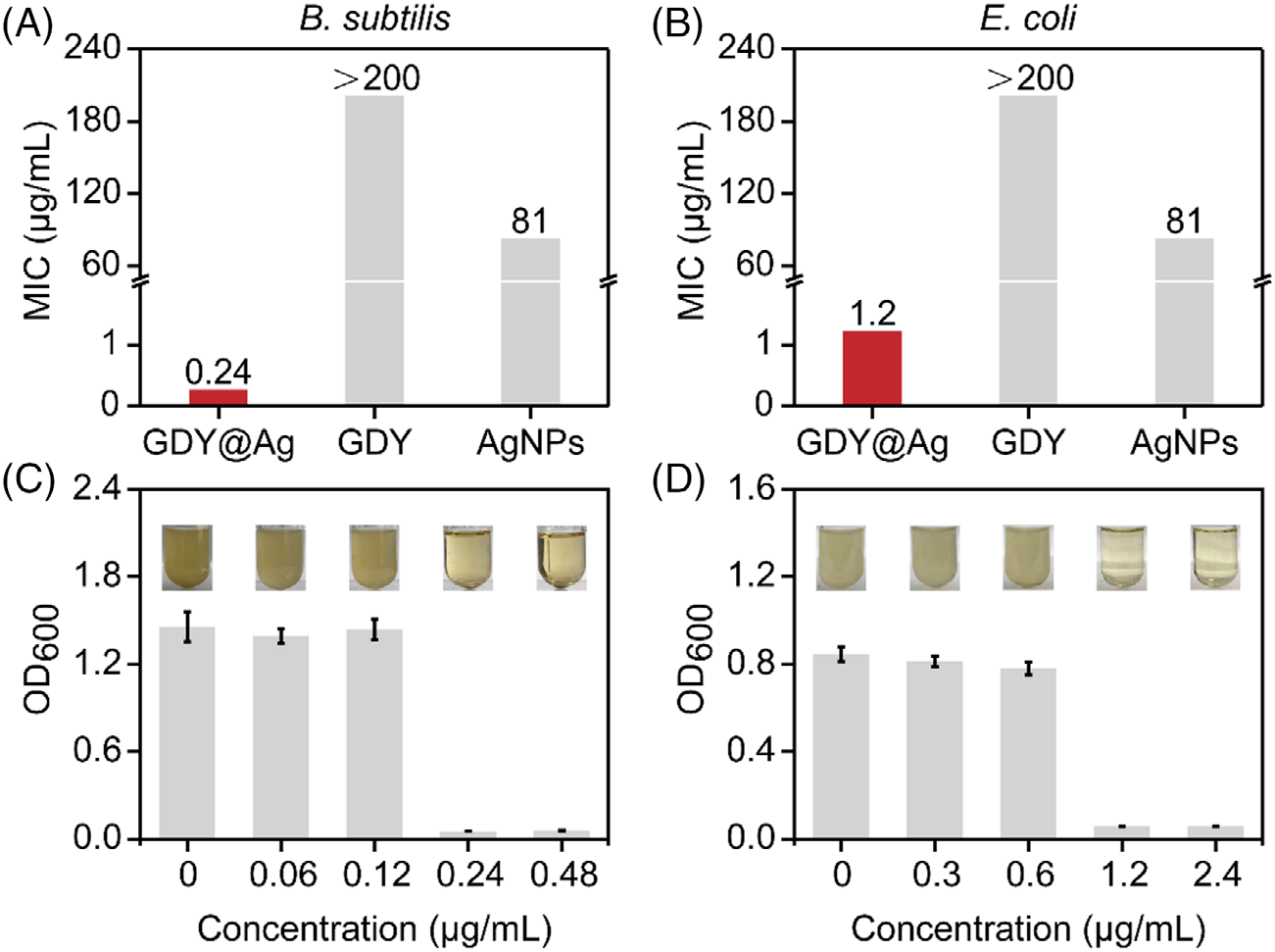
Antimicrobial capacity of GDY@Ag composite. (a and b) The minimum inhibitory concentration of GDY@Ag and its constituents. (c and d) GDY@Ag combats bacteria in a dose‐dependent manner. The insets are the digital turbidity images

Silver‐based materials are known for their prominent antibacterial properties. To verify the antimicrobial efficiency of GDY@Ag, we first determined its minimum inhibitory concentration (MIC) value based on the turbidity and the optical density at 600 nm of bacterial cultures. *Bacillus subtilis* (*B. subtilis*) and *Escherichia coli* (*E. coli*) were selected to represent gram‐positive and gram‐negative bacteria, respectively. As shown in Figure 3, the MIC of GDY@Ag towards *B. subtilis* was 0.24 μg/ml, and *E. coli* was 1.2 μg/ml. MIC for AgNPs was up to 81 μg/ml, yet GDY could not prevent the planktonic bacterial growth at the concentration of 200 μg/ml. The effect of CTAB and residual Ag^+^ on the antibacterial activity of the composites could be ignored due to their ultra‐low concentration (Figure S2). We carried out inductively coupled plasma mass spectrometry (ICP‐MS) to determine the accurate amount of Ag element contained in GDY@Ag. Calculations revealed that the element amount of Ag of GDY@Ag MIC towards *B. subtilis* and *E. coli* was about 0.14 and 0.7 μg/ml, respectively, demonstrating the improved antibacterial activity of GDY@Ag by at least two orders of magnitude compared with single AgNPs. This result was in agreement with previous work, where an anchor enhanced the stability of silver nanoparticles and together synergistically improved the antibacterial activity.^16^, ^40^

**FIGURE 4:**
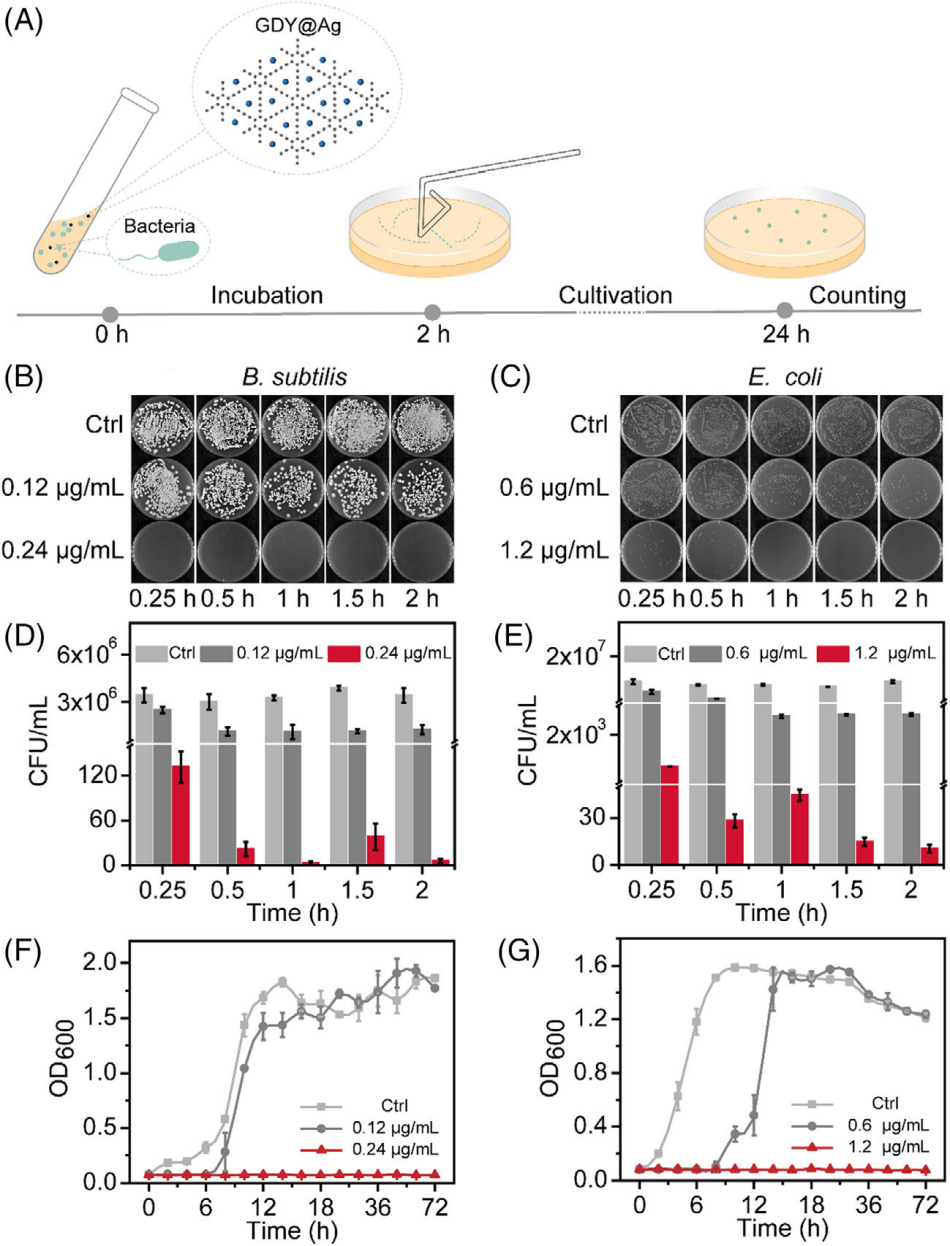
The short‐ and long‐term antibacterial effects of GDY@Ag. (a) Schematic of the antimicrobial analysis process. The short‐term inactivation efficiency of GDY@Ag towards different bacteria in 2 h was studied by plate counting method, (b and d) *B. subtilis*, (c and e) *E. coli*. Growth curve of two bacterial strains for 72 h, (f) *B. subtilis* and (g) *E. coli*

Next, we evaluated the instant bactericidal efficiency of GDY@Ag within 120 min using the conventional plate counting method (Figure 4a). As shown in Figure 4b–e, the composite at MIC killed 99.99% of bacteria within 15 min, demonstrating rapid antimicrobial activity. It is noticeable that the group of half MIC exhibits a partial inhibiting effect, which keeps the bacteria count at a steady state finally. The results revealed the dose‐dependent antibacterial property of GDY@Ag. Additionally, we studied the long‐term antibacterial activity of GDY@Ag by investigating the growth kinetics of bacteria for 3 days (Figures 4f,g). The obtained growth curve showed that the exponential phase of both two bacterial species lagged for several hours and finally reached the peak anyway in the case of half MIC, whilst the proliferation was totally inhibited by GDY@Ag at MIC (Figure 3). We could rationally infer that the remaining population in the half MIC case was temporarily tolerant that leads to the ultimate recovery, which calls the lagged phenomenon.^41^

### 3.3 Antimicrobial property of GDY@Ag

**FIGURE 5:**
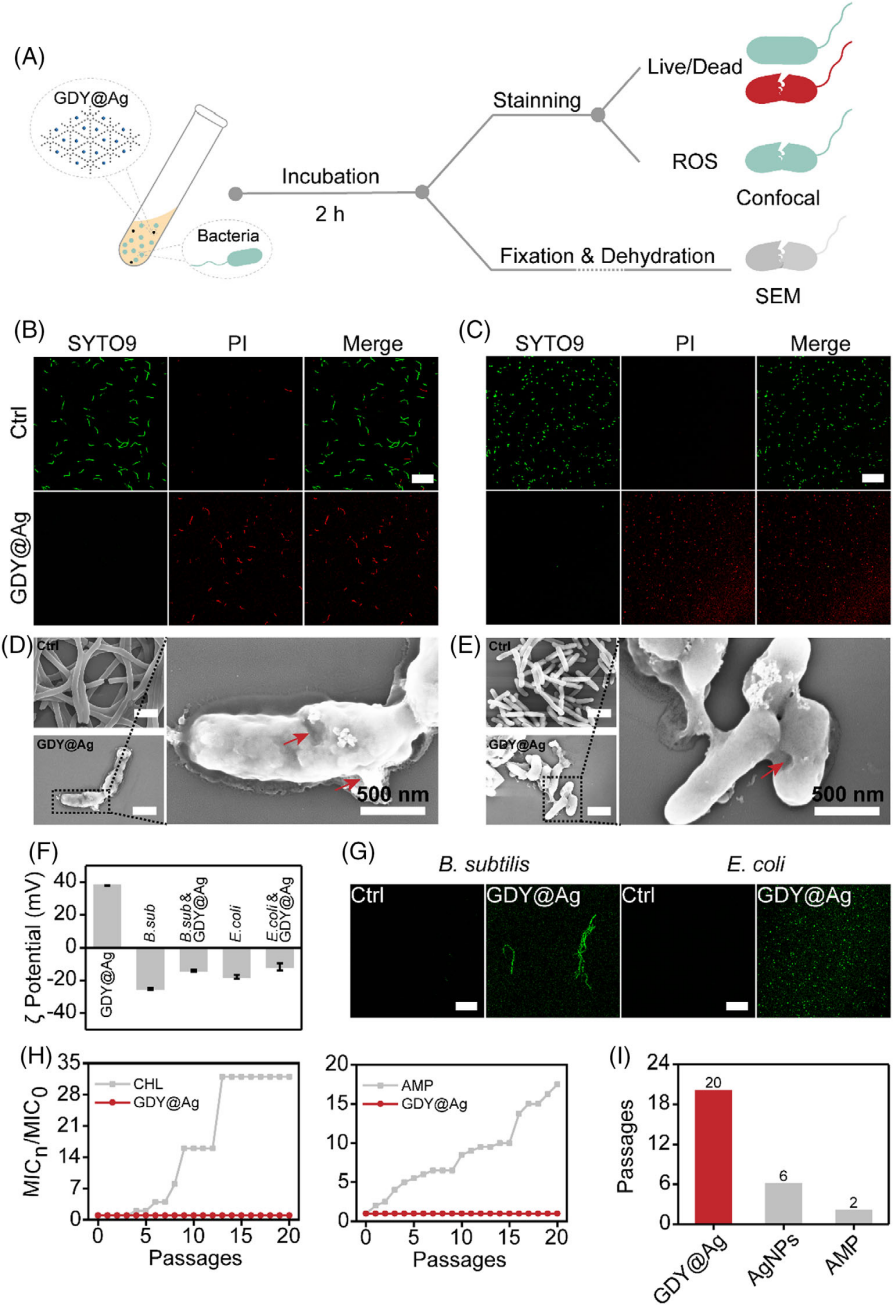
Physiological change of bacteria. (a) Schematic of the experimental protocol for antibacterial process and analysis. Live/dead staining images of *B. subtilis* (b) and *E. coli* (c) (Scale bar, 15 μm). SEM images of *B. subtilis* (d) and *E. coli* (e) (Scale bar, 1 μm). Red arrow, damaged cell membrane. (f) Zeta potential of GDY@Ag, bacteria, mixture of GDY@Ag and bacteria. (g) ROS level of bacteria. Bacteria treated with Milli‐Q water as control and GDY@Ag at MIC in all experiments. (h) The fold change of MIC after each of 20 consequent culture steps. *B. subtilis* (left), *E. coli* (right). (i) The first step of *E. coli* becoming resistant to bactericides. The data on AgNPs are from the literature^16^

After confirming the antibacterial property of GDY@Ag, we set out to explore the mechanism of the bactericidal activity (Figure 5a). According to the most discussed “insertion” mechanism of 2D carbon nanomaterials, we speculated that GDY@Ag inactivates bacteria by destroying their cell membrane. Consequently, we carried out the live/dead assay to determine whether the cell membrane is intact or not. Both *B. subtilis* (Figure 5b) and *E. coli* (Figure 5c) treated with GDY@Ag showed strong red fluorescence emitted by propidium iodide, whereas the control groups exhibited strong green fluorescence from SYTO9, revealing the substantial membrane disruption caused by GDY@Ag. Furthermore, we used SEM to observe the physical morphology changes of bacteria with and without GDY@Ag treatment. Compared to normal bacteria, both *B. subtilis* (Figure 5d) and *E. coli* (Figure 5e) exposure to GDY@Ag exhibited compromised membrane, suggesting the physical destructive effect of GDY@Ag towards bacteria. The zeta potential test showed that GDY@Ag was positively charged in aqueous solution (Figure 5f), which could enhance the electrostatic interaction between GDY@Ag and bacteria. In this case, GDY@Ag could easily capture bacteria and destroy their membrane. The morphological change of bacteria is commonly related to their oxidative state. Thus, we evaluated the reactive oxygen species (ROS) level using a probe, 2′,7′‐dichlorodihydrofluorescein diacetate (DCFH‐DA). Non‐fluorescent DCFH‐DA can freely diffuse into the cell and be hydrolysed by intracellular esterase and then be oxidized by ROS to form green fluorescent 2′,7′‐dichlorofluorescein (DCF). ROS level can be quantified by the fluorescence intensity of DCF. As shown in Figure 5g, two bacterial strains treated with GDY@Ag showed stronger green fluorescence than untreated, demonstrating that GDY@Ag induced the oxidative stress towards bacteria. These findings demonstrated that GDY@Ag kills bacteria through membrane damage and oxidative stress.

It is well known that the repeated use of antibiotics leads to the decreased susceptibility of bacteria, that is, bacteria develop drug resistance.^42^, ^43^ Our results demonstrated GDY@Ag inactivated bacteria by physical and oxidative damage, which differed from the mechanism of antibiotics,^4^ we thus hypothesized that GDY@Ag may not induce bacterial resistance. To test this hypothesis, we cultured 20 successive bacterial steps with two strains in media containing sub‐inhibitory GDY@Ag and antibiotics. In detail, planktonic bacteria were inoculated to the bactericides at half MIC, and the obtained generation was defined as the first passage; the subsequent passages were derived by treating the former one with its corresponding half MIC. As shown in Figure 5h, there was a 32‐fold change in the MIC of *B. subtilis* with chloramphenicol (CHL) treatment after 20 passages, the MIC of ampicillin (AMP) towards *E. coli* also increased 17.5 times, whilst the MIC did not change for the GDY@Ag treatment, indicating that GDY@Ag did not trigger the development of bacterial resistance. In the case of *E. coli*, it became resistant to ampicillin by the second step, but it did not develop resistance to GDY@Ag until the 20th (Figure 5i). Previous work reported that *E. coli* developed resistance to silver NPs after six cultivation steps.^16^ These results demonstrated that immobilized silver NPs possessed excellent bacterial susceptibility.

## 4 DISCUSSION

Graphdiyne (GDY) and graphdiyne oxide (GDYO) have evolved in several antibacterial studies recently. Graphdiyne‐modified TiO_2_ nanofibres have been prepared as titanium implants, avoiding bacterial infections through enhanced photocatalysis and prolonged antibacterial ability.^26^ GDY and GDYO could also kill bacteria through direct contact with bacteria, as well as ROS production by visible light illumination.^29^ Besides, other factors influencing the antimicrobial effect of GDY and GDYO against bacteria were assessed, such as material concentration, application time and shaking speed.^30^ These studies shed light on harnessing GDY and its derivatives as new alternatives for antibiotics. However, the TiO_2_ nanofibres would be too feeble to kill bacteria without ultraviolet irradiation. GDY and GDYO suppressed the growth of bacteria temporarily for 12 h at 1 mg/ml, and the antibacterial capacity of GDY could be negligible at 200 μg/ml. Hence, graphdiyne should be engaged with other effective bactericides to achieve great synergistic antisepsis. Herein, it is worth noting that to eliminate the side bactericidal effect of CTAB and the excess Ag^+^, we collected supernatant of each centrifugation and tested the corresponding antibacterial ability by the turbidity method. The fourth supernatant showed no inhibition of the bacterial growth (Figure S3); thus, a four‐time washing method was eventually determined in the process of GDY@Ag preparation, ensuring the individual antibacterial effect of GDY@Ag. Our findings showed the synergy of GDY with AgNPs could enhance the antibacterial ability. The MIC of GDY@Ag towards *B. subtilis* and *E. coli* was 0.24 and 1.2 μg/ml, respectively. These concentrations were far lower than the MIC of AgNPs (81 μg/mg) and GDY (> 200 μg/ml) against two bacterial strains. Moreover, we found that the antibacterial activity of GDY@Ag was stronger than that of Graphene @AgNPs reported yet.^44^, ^45^ This may be attributed to different positive charge densities on the surface of two nanocomposites. GDY@Ag was with higher positive charge density of about +38 ± 0.2 mV (Figure 5f) than GO‐AgNPs (+20.3 mV) reported, which strengthened its interaction with negatively charged bacteria.

Hitherto, the antibacterial mechanism of 2D carbon‐based materials research refers to the following two mechanisms: (1) insertion of the sharp edge into the bacterial membrane, leading to the extraction of cellular contents,^46^, ^47^ (2) oxidative stress injury induced by excessive elevated ROS production.^48^, ^49^, ^50^ The antimicrobial capacity of GDY is considered as a result of “physical” effects in combination with “chemical” actions.^30^ The positively charged GDY is favourable to wrap bacterial surface with negative charge, which may prevent bacterial growth. With the increase in their interaction, GDY nanosheets can directly insert and disrupt the bacterial membranes. The zeta potential revealed that positively charged GDY@Ag contacted with negatively charged bacteria through electrostatic interaction (Figure 5f). The bacterial membrane destroyed by GDY@Ag was observed by SEM images (Figure 5d). Oxidative stress induced by GDY has a negligible effect on bactericidal activity without irradiation.^29^, ^30^ As we know, Ag^+^ released from AgNPs and oxidative stress induced by it are two major antimicrobial factors.^51^ Silver ions released from GDY@Ag were not detected by ICP‐MS (Figure S4), which could exclude the role of Ag ions in antibacterial activity. In fact, the level of cellular ROS significantly improved when bacteria exposed to GDY@Ag (Figure 5g), which suggested that oxidative stress induced by AgNPs played a crucial role in GDY@Ag against bacteria. Overall, the antibacterial mechanism of GDY@Ag may be contributed to a synergistic action of GDY and AgNPs. GDY@Ag firstly wrapped bacteria through electrostatic interaction, and then “insertion mode” and oxidative stress combined to destroy the cell membrane to bacterial death.

In addition, our study opened the door to harnessing 2D materials to stabilize metal nanoparticles as the last ditch for combatting the evolving bacteria, preventing unexpected metal resistance. Previous solutions to stabilize metal nanoparticles refer to polymers, protein inhibitors; for instance, pomegranate rind extract could inhibit flagellin production which mediates the aggregation of AgNPs.^16^ GDY@Ag also immobilizes AgNPs; the passive aggregation of AgNPs was totally inhibited in this case which preserves bacterial susceptibility. GDY@Ag neither binds any target inside bacteria nor gets inside bacteria directly, which is totally different from antibiotics; in this case, it is difficult for bacteria to develop resistance towards GDY@Ag in a short time. Despite this, organisms are slowly but constantly evolving; physical damage seems to be effective to guard against resistance for the time being. Since we cannot anticipate the future, it is necessary to postpone the adaption of resistance to arm ourselves with various antibacterial agents. Besides, it is very important for bactericides to possess good biocompatibility in practical applications. Our results found that the concentration of 2.4‐μg/ml GDY@Ag only caused less than 5% human‐derived normal mammary epithelial cell line MCF‐10A death (Figure S5), showing an outstanding application potential.

## CONFLICT OF INTEREST

The authors declare no conflict of interest.

## AUTHOR CONTRIBUTIONS

Min Lv and Simin Qin conceived and designed the study. Simin Qin and Mo Xie performed the experiments. Min Lv and Simin Qin co‐wrote the paper. All authors analysed and discussed the results and agreed to publish the manuscript.

## Supporting information


Figure S1‐S5

## Data Availability

Data sharing is not applicable to this article as no new data were created or analyzed in this study.
