# Supplementary material for: Insight into the antibacterial resistance of graphdiyne functionalized by silver nanoparticles
Source: Cell Prolif. 2022 May 3;55(5):e13236. doi: 10.1111/cpr.13236 (PMC9136490; doi:10.1111/cpr.13236)
Supplement: Supplementary file 1 — Figure S1‐S5 [file CPR-55-e13236-s001.docx]

**Supporting Information**

**Insight into the Antibacterial Resistance of Graphdyine Functionalized by Silver Nanoparticles**

Simin Qin^1,4^, Mo Xie^6^, Shuting Cao^1,4^, Jiang Li^1,3^, Lihua Wang^1,3^, Shi-Hua Luo^5*^, Min Lv^2,3*^

1. Division of Physical Biology, CAS Key Laboratory of Interfacial Physics and Technology, Shanghai Institute of Applied Physics, Chinese Academy of Sciences, Shanghai 201800, China.

2. College of Chemistry and Materials Science, Shanghai Normal University, Shanghai, 200234 China.

3. The Interdisciplinary Research Center, Shanghai Synchrotron Radiation Facility, Zhangjiang Laboratory, Shanghai Advanced Research Institute, Chinese Academy of Sciences, Shanghai, 201210, China.

4. University of Chinese Academy of Sciences, Beijing 100049, China.

5. Department of Traumatology, Rui Jin Hospital, School of Medicine, Shanghai Jiao Tong University, Shanghai, 200025, China.

6. State Key Laboratory of Organic Electronics and Information Displays & Jiangsu Key Laboratory for Biosensors, Institute of Advanced Materials (IAM), Jiangsu National Synergetic Innovation Center for Advanced Materials (SICAM), Nanjing University of Posts and Telecommunications, Nanjing 210023, China.

Simin Qin and Mo Xie equally contributed to this work.

*Corresponding Authors: [jqab@163.com](mailto:jqab@163.com); lvmin@shnu.edu.cn

**Methods**

**MTT assay** Human derived normal mammary epithelial cell Michigan Cancer Foundation 10A (MCF-10A) was cultured 24-well cell culture plates overnight. Then, GDY@Ag at serial concentration diluted with cell medium was added into the plates to treat cells for 2 h. Subsequently, all the supernatant was removed. 500 μL 1×PBS was used to wash the wells twice. MTT was diluted with cell medium and added to the plate. After 4 h incubation, 10 % acidifying Sodium dodecyl sulfate (SDS) was added to the plate and incubated overnight. Cell viability was finally determined by measuring OD_570_.

**
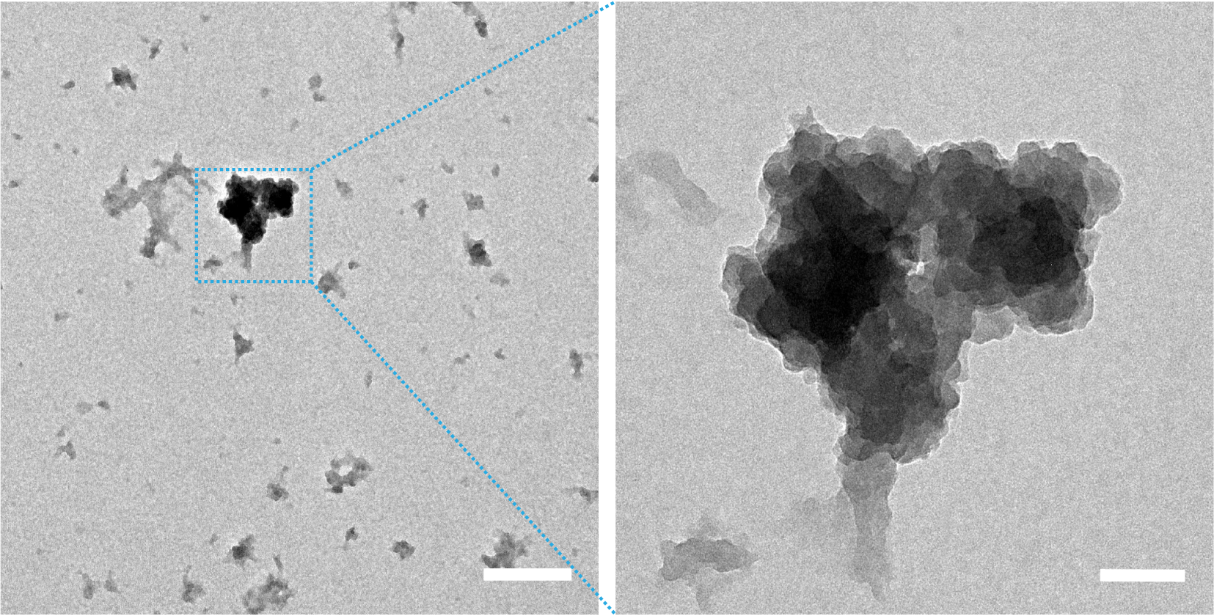
**

**Figure S1.** TEM images of GDY sheets (Scale bar, 0.5 μm), the right ones are the enlarged image of the part in blue box (Scale bar, 100 nm).


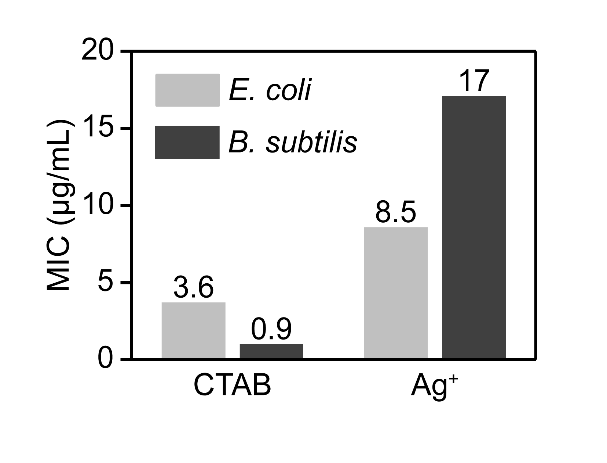


**Figure S2.** MIC of CTAB and Ag^+^ towads *B. subtilis* and *E. coli*.


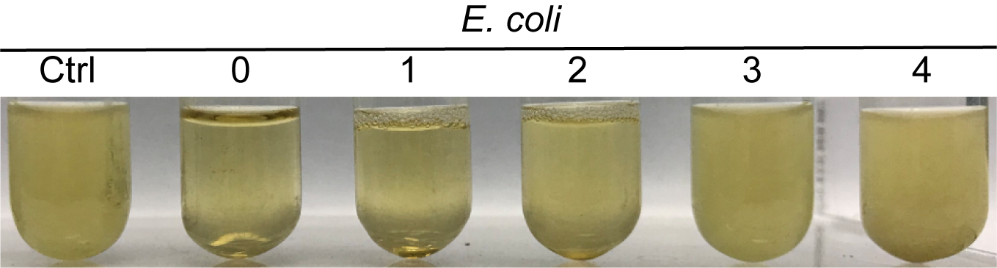


**Figure S3.** Antibacterial activity of four washing supernatants.

**
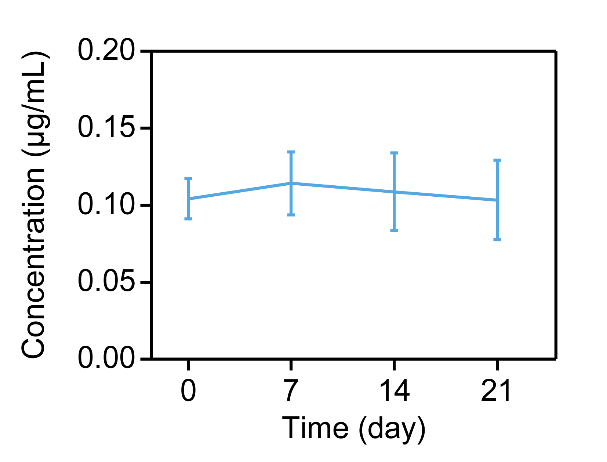
**

**Figure S4.** Residual silver ion in GDY@Ag (240 μg/mL) solution within 3 weeks.





**Figure S5.** The viability of MCF-10A cells exposure to GDY@Ag for 2 h.
